# A nomogram for individualized estimation of survival among adult patients with adrenocortical carcinoma after surgery: a retrospective analysis and multicenter validation study

**DOI:** 10.1186/s40880-019-0426-0

**Published:** 2019-11-27

**Authors:** Jianqiu Kong, Junjiong Zheng, Jinhua Cai, Shaoxu Wu, Xiayao Diao, Weibin Xie, Xiong Chen, Chenyi Liao, Hao Yu, Xinxiang Fan, Chaowen Huang, Zhuowei Liu, Wei Chen, Qiang Lv, Haide Qin, Jian Huang, Tianxin Lin

**Affiliations:** 1 Department of Urology, Sun Yat-Sen Memorial Hospital, Sun Yat-Sen University, 107 Yan Jiang West Road, Guangzhou, 510120 Guangdong P. R. China; 2 Guangdong Provincial Key Laboratory of Malignant Tumor Epigenetics and Gene Regulation, Sun Yat-Sen Memorial Hospital, Sun Yat-Sen University, Guangzhou, 510120 Guangdong P. R. China; 3 Department of Neurology, Sun Yat-Sen Memorial Hospital, Sun Yat-Sen University, Guangzhou, 510120 Guangdong P. R. China; 4 Department of Obstetrics and Gynecology, Third Affiliated Hospital of Sun Yat-sen University, Guangzhou, 510630 Guangdong P. R. China; 5 Department of Urology, Sun Yat-sen University Cancer Center, Guangzhou, 510060 Guangdong P. R. China; 6 Department of Urology, First Affiliated Hospital of Sun Yat-sen University, Guangzhou, 510080 Guangdong P. R. China; 7 Department of Urology, The First Affiliated Hospital of Nanjing Medical University, Nanjing, 210029 Jiangsu P. R. China; 8 State Key Laboratory of Oncology in South China, Guangzhou, 510120 Guangdong P. R. China

**Keywords:** Adrenocortical carcinoma, Adult patients, Overall survival, Nomogram, Validation, Decision curve analysis, Surveillance Epidemiology, and End Results (SEER), The Cancer Genome Atlas (TCGA), Multicenter

## Abstract

**Background:**

Clinical outcome of adrenocortical carcinoma (ACC) varies because of its heterogeneous nature and reliable prognostic prediction model for adult ACC patients is limited. The objective of this study was to develop and externally validate a nomogram for overall survival (OS) prediction in adult patients with ACC after surgery.

**Methods:**

Based on the data from the Surveillance Epidemiology, and End Results (SEER) database, adults patients diagnosed with ACC between January 1988 and December 2015 were identified and classified into a training set, comprised of 404 patients diagnosed between January 2007 and December 2015, and an internal validation set, comprised of 318 patients diagnosed between January 1988 and December 2006. The endpoint of this study was OS. The nomogram was developed using a multivariate Cox proportional hazards regression algorithm in the training set and its performance was evaluated in terms of its discriminative ability, calibration, and clinical usefulness. The nomogram was then validated using the internal SEER validation, also externally validated using the Cancer Genome Atlas set (TCGA, 82 patients diagnosed between 1998 and 2012) and a Chinese multicenter cohort dataset (82 patients diagnosed between December 2002 and May 2018), respectively.

**Results:**

Age at diagnosis, T stage, N stage, and M stage were identified as independent predictors for OS. A nomogram incorporating these four predictors was constructed using the training set and demonstrated good calibration and discrimination (C-index 95% confidence interval [CI], 0.715 [0.679–0.751]), which was validated in the internal validation set (C-index [95% CI], 0.672 [0.637–0.707]), the TCGA set (C-index [95% CI], 0.810 [0.732–0.888]) and the Chinese multicenter set (C-index [95% CI], 0.726 [0.633–0.819]), respectively. Encouragingly, the nomogram was able to successfully distinguished patients with a high-risk of mortality in all enrolled patients and in the subgroup analyses. Decision curve analysis indicated that the nomogram was clinically useful and applicable.

**Conclusions:**

The study presents a nomogram that incorporates clinicopathological predictors, which can accurately predict the OS of adult ACC patients after surgery. This model and the corresponding risk classification system have the potential to guide therapy decisions after surgery.

## Background

Adrenocortical carcinoma (ACC) is a rare disease in both pediatric and adult patients with an overall incidence of 0.5–2.0 cases per million people per year [1]. Complete surgical resection is considered as the main curative form of treatment for localized ACC [2]. However, ACC is a vicious tumor with a high degree of malignancy and recurrence rate [3–6]. Its 5-year overall survival (OS) rate is estimated to range between 16 and 60% [7–9].

Adjuvant therapy including mitotane has demonstrated the potential to improve the prognosis of ACC patients [10–12], although confirmatory randomized, prospective trials on adjuvant therapy are yet to be published. If the prognosis of ACC could be accurately predicted, comprehensive treatment would be timely given to high-risk patients to improve their survival outcome. The American Joint Committee on Cancer (AJCC) TNM staging system is globally recognized and implemented to estimate the survival of ACC patients [13, 14], but it is largely constrained by its inability to consider other determining clinicopathological factors, such as age, gender, and tumor size, which may also have considerable impact on the patients’ survival [15, 16].

Only a few studies have established prediction models for clinicians and researchers to access the prognosis of ACC patients because of its low incidence [17–20]. However, these studies were partly limited for clinical applicability as in some, the patient’s age was used as a categorical variable rather than continuous variable [17, 18], in others, the cases with insufficient data (data of radiation therapy, chemotherapy, and histologic grade et al.) were not excluded for analysis or even lacked external validation [17, 20]. In addition, another important determining limitation was that these proposed models were developed using the data of ACC patients of all ages and thereby neglected the differences in prognosis predictors between pediatric and adult patients [17–20]. In fact, adult and pediatric ACC patients are different not only in incidence and clinical presentation but also in some aspects of biological behaviors. ACC in adult patients is more aggressive and is associated with poorer clinical outcomes despite undergoing complete surgical resection as compared to pediatrics ACC. The 5-year survival rate in adult patients was reported of being 37%–39% and in pediatric patients 53%–56% [21, 22]. One study analyzing the data from the Surveillance, Epidemiology and End Results (SEER) database found that the overall 5-year survival of ACC patients in adults was mediocrely between 30 and 40% while that of pediatrics was 57% [23]. The genomic characteristics of ACC are also different between pediatric and adult patients. For instance, germline *TP53* mutations are less common in adults with ACC, and IGF2 overexpression is a marker of poor prognosis in adult ACC patients, but not in pediatric patients [24, 25]. Meanwhile, adult patients seem to have less obvious symptoms of hormonal overproduction, i.e. virilization and precocious puberty, and have clear-cut pathological criteria for malignancy [26] meaning that tumors among adult patients can be adequately classified based on the Weiss or Van Slooten scores. As such, an easy-to-implement model for prognostication of the postoperative survival tailored for adult ACC patients is greatly needed to provide more personalized treatment, especially for high-risk patients.

In the present study, we aimed to develop a nomogram for predicting the survival of post-operative adult ACC patients using the SEER database and to validate it using external validation using the Cancer Genome Atlas (TCGA) database and a multicenter Chinese cohort for wider clinical application.

## Methods

### Patients and data collection

**Fig. 1:**
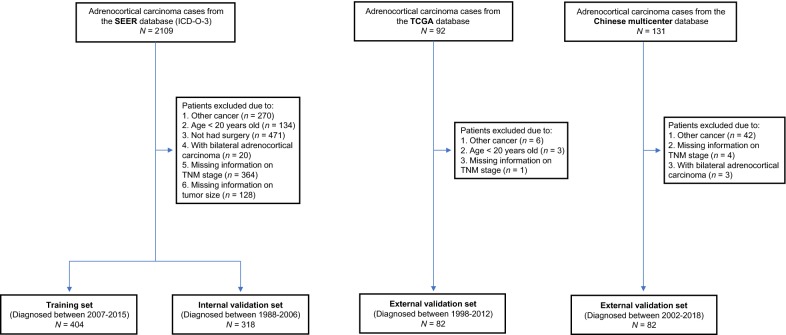
Flowchart illustrating patient selection for this study

In this multicenter retrospective study, three independent datasets of adult ACC patients were retrieved. The cases recruitment methodology is illustrated in Fig. 1. The inclusion criteria for data extraction were (1) pathology-confirmed ACC diagnosis; (2) patients aged ≥ 20 years who underwent surgery at the primary tumor site; and (3) availability of complete clinicopathological and follow-up data. The exclusion criteria were (1) patients with other synchronous cancers or prior diagnosis with other tumors; (2) patients with bilateral ACC. Ultimately, eligible ACC patients from the SEER database (January 1988- to December 2015, ICD-O-3) were identified and classified as the SEER training set (diagnosed between January 2007 and December 2015) and the SEER internal validation set diagnosed between January 1988 and December 2006). In addition, two other independent datasets comprising of the TCGA validation set (TCGA-ACC project, diagnosed between 1998 and 2012) and a Chinese multicenter validation set (diagnosed between December 2002 and May 2018 from four hospitals, namely the Sun Yat-sen Memorial Hospital, the First Affiliated Hospital of Sun Yat-sen University, Sun Yat-sen University Cancer Center and Jiangsu Province Hospital) were used for external validation. For the Chinese cohort, the retrospective analysis of anonymous patient data was approved by the institutional review board at each participating institution. Due to the retrospective nature of this study, informed consent was not required and patients’ data were used anonymously.

Demographic and clinicopathological data including age at diagnosis, gender, tumor laterality, tumor size, TNM stage, tumor stage group, survival status, and survival time were retrieved. TNM stage was defined according to the UICC/AJCC TNM Classification. The tumor stage group was defined based on the 7th AJCC staging system and the European Network for the Study of Adrenal Tumors (ENSAT) staging system consistent with the 8th AJCC staging system. The main outcome was OS, defined as the time from the date of diagnosis to the date of death or last follow-up.

Of note, the SEER data were accessed using the SEER*Stat version 8.3.5 software on January 3, 2019, and data from the TCGA set were downloaded from the TCGA-ACC project on January 23, 2019 (https://portal.gdc.cancer.gov/). For the Chinese cohort, the data were censored on December 31, 2018.

### Development of the nomogram

In the training set, clinicopathological predictors were tested using the univariable Cox proportional hazards regression analyses. Three models for OS prediction using multivariable Cox proportional hazards regression analyses were developed. Model 1 incorporated the TNM stage, while models 2 and 3 incorporated the 7th AJCC stage group and ENSAT stage group, respectively. Backward stepwise selection was applied by using the Akaike’s Information Criterion (AIC) as the stopping rule [27] and age at diagnosis was included in all three models. The discrimination accuracy of the models was quantified using the Harrell’s concordance index (C-index) [28]. The optimal model was selected by comparing their C-indices and based on which the nomogram was developed.

### Performance assessment of the nomogram in the training set

C-index was obtained to quantitatively evaluate the discriminative ability of the nomogram. Calibration curves were plotted to assess the calibration of the nomogram. Bootstrapping using 1000 resampling procedures was applied to calculate the C-index that was corrected for potential overfitting.

### Validation of the nomogram

The performance of the nomogram was validated using the SEER internal validation dataset and externally validated using the TCGA and Chinese dataset. The multivariate Cox proportional hazards regression formula of the nomogram formed in the training set was applied to the patients in the validation sets, with risk scores calculated for each patient to reflect the risk of cancer mortality. Cox proportional hazards regression analyses were performed using the risk scores in the validation sets. The discrimination and calibration of the nomogram were then assessed based on the regression analyses to validate its performance.

### Survival risk classification based on the nomogram

In the training dataset, the optimal risk score for ACC mortality cutoff value was identified using the X-tile plots [29]. Based on the value obtained, all patients were classified into a high- and low-risk group. The Kaplan–Meier method and log-rank test were used to assess and compare the OS of adult ACC patients after surgery in the different risk groups. Stratified analyses were also performed within the various subgroups according to sex and tumor location.

### Clinical usefulness of the nomogram

Decision curve analysis (DCA) was performed by calculating the net benefits for a range of threshold probabilities to estimate the clinical usefulness of the nomogram. The DCA algorithm, a validated approach, was utilized for evaluating alternative diagnostic and prognostic strategies [30].

### Statistical analysis

The X-tile software version 3.6.1 (Yale University, New Haven, CT, USA) was used to determine the optimal risk score cutoff value. All other computations were conducted using the R software, version 3.5.2 (The R Foundation for Statistical Computing, https://www.r-project.org/). The Cox proportional hazards regression analyses were performed by the R software “survival” and “MASS” packages. The nomogram and calibration plots were produced using the “rms” package. The DCA was performed using the function “stdca.R”. Statistical significance was set at *P* values less than 0.05 in a two-tailed test.

## Results

### Patient characteristics

In total, 722 eligible ACC patients from the SEER database were identified and classified as the SEER training set (*n* = 404) and the SEER internal validation set (*n *= 318). There were also two external validation sets, namely the TCGA validation set (*n* = 82) and the Chinese multicenter validation set (*n* = 82). The patients’ characteristics of the training and three validation datasets are shown in Table 1. The median follow-up of the entire dataset was 51 months (interquartile ranges [IQR], 45–57 months) for the training dataset; 167 months (IQR, 156–178 months) for the internal validation dataset; 61 months (IQR, 42–80 months) for the TCGA validation set; and 22 months (IQR, 15–29 months) for the Chinese multicenter validation set. Furthermore, the generalized 5-year OS of these datasets was also calculated. In the SEER training dataset, the 5-year OS was 40.4% (95% confidence interval [CI], 34.6%–46.1%). For the validation datasets, the 5-year OS was 41.1% (95% CI 35.7%–46.6%), 60.4% (95% CI 47.9%–72.9%) and 63.6% (95% CI 48.7%–78.5%) for the SEER internal validation, TCGA and Chinese multicenter validation set, respectively.

**Table 1:** Baseline characteristics of the investigated patients as per different cohorts (*N* = 886)

Characteristics	Training set (*n *= 404)			Internal validation set (*n *= 318)			TCGA validation set (*n *= 82)			Chinese multicenter validation set (*n *= 82)		
	Number of patients	Low risk (%)	High risk (%)	Number of patients	Low risk (%)	High risk (%)	Number of patients	Low risk (%)	High risk (%)	Number of patients	Low risk (%)	High risk (%)
Age, years												
Median (IQR)	54 (20–89)	55 (20–89)	61 (27–86)	52 (20–85)	55 (20–85)	59 (34–74)	50 (20–83)	48 (20–83)	59 (23–71)	49 (19–79)	48 (19–77)	61 (49–79)
Sex												
Male	146	128 (87.7%)	18 (12.3%)	133	125 (94.0%)	8 (6.0%)	28	26 (92.9%)	2 (7.1%)	38	34 (89.5%)	4 (10.5%)
Female	258	232 (89.9%)	26 (10.1%)	185	169 (91.4%)	16 (8.6%)	54	47 (87.0%)	7 (13.0%)	44	42 (95.5%)	2 (4.5%)
Tumor location												
Left	224	204 (91.1%)	20 (8.9%)	163	150 (92.0%)	13 (8.0%)	43	37 (86.0%)	6 (14.0%)	56	53 (94.6%)	3 (5.4%)
Right	180	156 (86.7%)	24 (13.3%)	155	144 (92.9%)	11 (7.1%)	39	36 (92.3%)	3 (7.7%)	26	23 (88.5%)	3 (11.5%)
Tumor size, cm												
Median (IQR)	11.0 (1.2–80.0)	10.7 (1.2–80.0)	11.8 (2.6–21.0)	11.0 (1.2–34.0)	11.0 (1.2–34.0)	12.3 (1.7–22.5)	–	–	–	9.1 (0.8–17.8)	9.5 (0.8–17.8)	6.5 (4.5–11.1)
7th AJCC T stage												
T1	26	26 (100.0%)	0 (0.0%)	16	16 (100.0%)	0 (0.0%)	7	7 (100.0%)	0 (0.0%)	6	6 (100.0%)	0 (0.0%)
T2	194	192 (99.0%)	2 (1.0%)	190	189 (99.5%)	1 (0.5%)	45	43 (95.6%)	2 (4.4%)	38	38 (100.0%)	0 (0.0%)
T3	109	90 (82.6%)	19 (17.4%)	58	49 (84.5%)	9 (15.5%)	11	10 (90.9%)	1 (9.1%)	20	19 (95.0%)	1(5.0%)
T4	75	52 (69.3%)	23 (30.7%)	54	40 (74.1%)	14 (25.9%)	19	13 (68.4%)	6 (31.6%)	18	13 (72.2%)	5 (17.8%)
7th AJCC N stage												
N0	371	351 (94.6%)	20 (5.4%)	290	286 (98.6%)	4 (1.4%)	73	71 (97.3%)	2 (2.7%)	74	72 (97.3%)	2 (2.7%)
N1	33	9 (27.3%)	24 (72.7%)	28	8 (28.6%)	20 (71.4%)	9	2 (22.2%)	7 (77.8%)	8	4 (50.0%)	4 (50.0%)
7th AJCC M stage												
M0	328	319 (97.3%)	9 (2.7%)	287	277 (96.5%)	10 (3.6%)	66	66 (100.0%)	0 (0.0%)	68	68 (100.0%)	0 (0.0%)
M1	76	41 (53.9%)	35 (46.1%)	31	17 (54.8%)	14 (45.2%)	16	7 (43.8%)	9 (56.2%)	14	8 (57.1%)	6 (42.9%)
7th AJCC stage group												
I	24	24 (100.0%)	0 (0.0%)	15	15 (100.0%)	0 (0.0%)	7	7 (100.0%)	0 (0.0%)	5	5 (100.0%)	0 (0.0%)
II	173	173 (100.0%)	0 (0.0%)	176	176 (100.0%)	0 (0.0%)	41	41 (100.0%)	0 (0.0%)	31	31 (100.0%)	0 (0.0%)
III	83	83 (100.0%)	0 (0.0%)	52	52 (100.0%)	0 (0.0%)	11	11 (100.0%)	0 (0.0%)	22	22 (100.0%)	0 (0.0%)
IV	124	80 (64.5%)	44 (35.5%)	75	51 (68.0%)	24 (32.0%)	23	14 (60.9%)	9 (39.1%)	24	18 (75.0%)	6 (25.0%)
ENSAT stage group												
I	24	24 (100.0%)	0 (0.0%)	15	15 (100.0%)	0 (0.0%)	7	7 (100.0%)	0 (0.0%)	5	5 (100.0%)	0 (0.0%)
II	173	173 (100.0%)	0 (0.0%)	176	176 (100.0%)	0 (0.0%)	41	41 (100.0%)	0 (0.0%)	31	31 (100.0%)	0 (0.0%)
III	131	122 (93.1%)	9 (6.9%)	96	86 (89.6%)	10 (10.4%)	0 (0.0%)	0 (0.0%)	0 (0.0%)	0 (0.0%)	0 (0.0%)	0 (0.0%)
IV	76	41 (53.9%)	35 (46.1%)	31	15 (48.4%)	16 (51.6%)	34	25 (73.5%)	9 (26.5%)	46	40 (87.0%)	6 (23.0%)

Data are n or n (%) unless indicated otherwise. The ENSAT staging system was consistent with the 8th AJCC staging system

*IQR* interquartile range, *TCGA* the Cancer Genome Atlas, *AJCC* the American Joint Committee on Cancer, *ENSAT* European Network for the Study of Adrenal Tumors

### Development of the nomogram and performance assessment

**Fig. 2:**
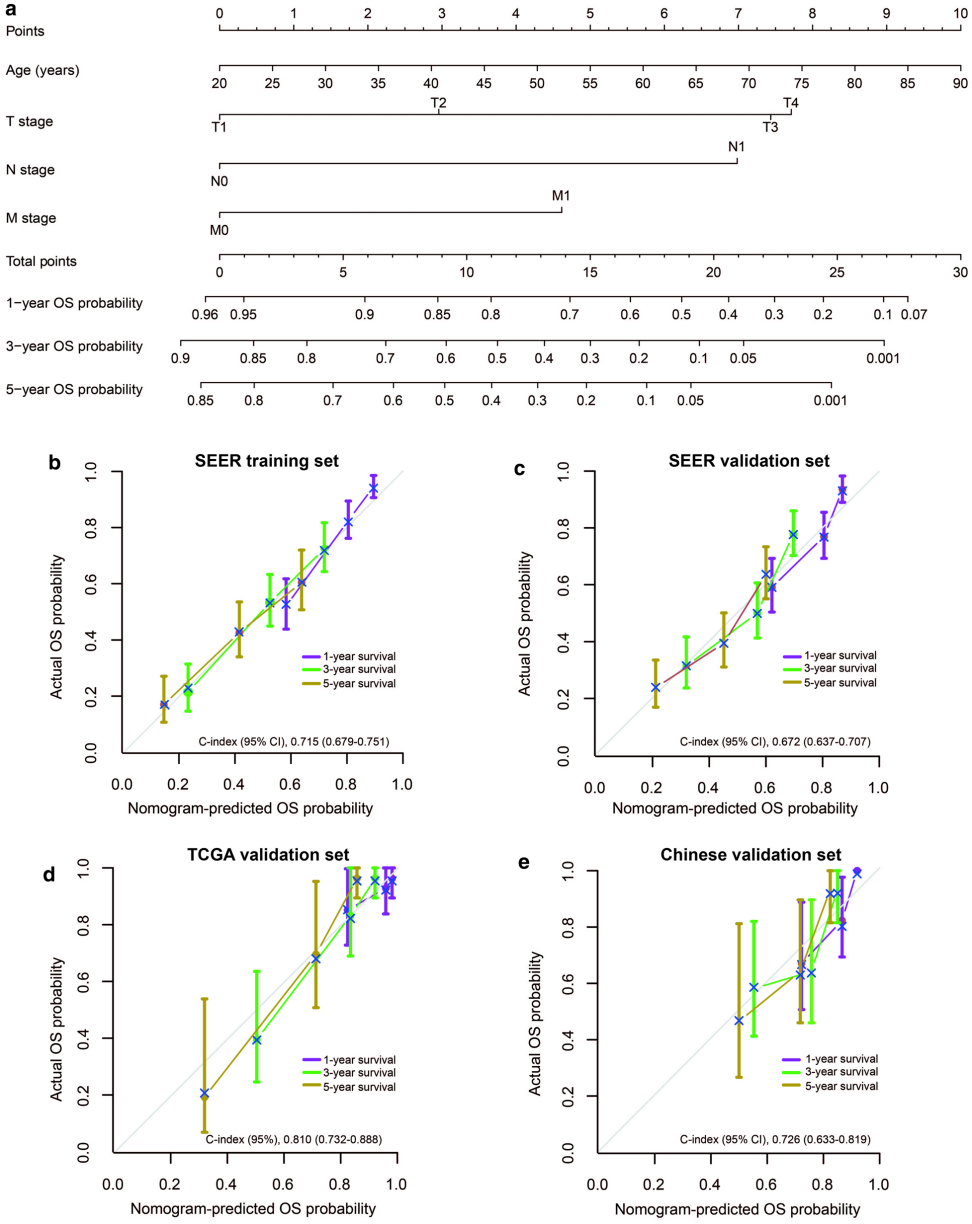
The formulated nomogram and its calibration plots. **a** This nomogram enables the prognostication of the 1-, 3- and 5-year estimates of the OS of ACC patients after surgery. Calibration plots of the nomogram performed in the **b** SEER training, **c** SEER internal validation, **d** the TCGA validation and **e** the Chinese multicenter validation set, respectively. Nomogram-predicted OS is plotted on the *x*-axis; actual OS is plotted on the y-axis. Dots represent nomogram-predicted probabilities. An ideal prediction would correspond to the diagonal 45° gray line slope of **b**–**e**. The score range of the nomogram is 0 to 29.3. *OS* overall survival, *ACC* adrenocortical carcinoma, *SEER* the Surveillance Epidemiology, and End Results database, *TCGA* the Cancer Genome Atlas set

Table 2 shows the findings of univariate and multivariate analyses in the training set. Age at diagnosis, ENSAT stage group, and 7th AJCC T, N, M and TNM stage were found to be significantly associated with OS. As for the multivariate analyses, age at diagnosis was included in all three models. Model 1 incorporated T stage, N stage, and M stage, while models 2 and 3 incorporated the 7th AJCC TNM and ENSAT stage group, respectively. The Cox regression coefficients of each included factors in the three models are displayed in Table 3. The C-indices of the models are listed in Table 4. Model 1 demonstrated the superior discrimination power in predicting OS (C-index [95% CI], 0.715 [0.679–0.751]) compared with model 2 and 3. Thence, model 1 was chosen as the optimal model, and a nomogram was developed on the basis of its regression result (Fig. 2a). The calibration curves for the 1-, 3- and 5-year OS showed favorable calibration of the nomogram in the training set (Fig. 2b).

**Table 2:** Univariate and multivariate Cox regression analyses of clinicopathologic factors with overall survival in the SEER training set

Characteristics	Univariable analyses		Model 1 Multivariable analyses		Model 2 Multivariable analyses		Model 3 Multivariable analyses	
	HR (95%CI)	*P*	HR (95% CI)	*P*	HR (95% CI)	*P*	HR (95% CI)	*P*
Age (continuous)	1.017 (1.008–1.027)	< 0.001*	1.021 (1.012–1.031)	< 0.001*	1.018 (1.008–1.027)	< 0.001*	1.019 (1.009–1.028)	< 0.001*
Sex (male *vs*. female)	0.822 (0.617–1.096)	0.182	–	–	–	–	–	–
Tumor location (left *vs.* right)	0.981 (0.742–1.297)	0.892	–	–	–	–	–	–
Tumor size (continuous)	1.012 (0.993–1.032)	0.227	–	–	–	–	–	–
7th AJCC T stage		< 0.001*		< 0.001*				
T1	Reference		Reference	–	–	–	–	–
T2	1.521 (0.734–3.154)	0.260	1.544(0.743–3.208)	0.244	–	–	–	–
T3	3.412 (1.636–7.116)	0.001*	2.981 (1.428–6.225)	0.004*	–	–	–	–
T4	4.364 (2.063–9.233)	< 0.001*	3.105 (1.454–6.633)	0.003*	–	–	–	–
7th AJCC N stage (N0 *vs.* N1)	3.448 (2.370–5.341)	< 0.001*	2.789 (1.801–4.319)	< 0.001*	–	–	–	–
7th AJCC M stage (M0 *vs.* M1)	2.773 (2.019–3.808)	< 0.001*	1.970 (1.391–2.791)	< 0.001*	–	–	–	–
7th AJCC TNM stage		< 0.001*				< 0.001*		
I	Reference		–	–	Reference		–	–
II	1.297 (0.622–2.705)	0.489	–	–	1.196 (0.572–2.498)	0.634	–	–
III	2.599 (1.225–5.515)	0.013*	–	–	2.375 (1.117–5.049)	0.025*	–	–
IV	4.097 (1.976–8.498)	< 0.001*	–	–	3.897 (1.878–8.087)	< 0.001*	–	–
ENSAT stage group		< 0.001*						
I	Reference		–	–	–	–	Reference	< 0.001*
II	1.297 (0.622–2.706)	0.488	–	–	–	–	1.191 (0.570–2.489)	0.642
III	2.782 (1.339–5.779)	0.006*	–	–	–	–	2.545 (1.223–5.299)	0.013*
IV	4.891 (2.317–10.322)	< 0.001*	–	–	–	–	4.752 (2.251–10.034)	< 0.001*

*TCGA* the Cancer Genome Atlas, *AJCC* the American Joint Committee on Cancer, *ENSAT* European Network for the Study of Adrenal Tumors, *HR* Hazard Ratio, *CI* confidence interval

*P < 0.05

**Table 3:** The Cox regression coefficients of the three models of the SEER training set

Model and variable	Cox regression coefficient
Model 1	
Age	0.0210
7th AJCC T stage	
T1	Reference
T2	0.4343
T3	1.0923
T4	1.1331
7th AJCC N stage	1.0257
7th AJCC M stage	0.6781
Model 2	
Age	0.0176
7th AJCC stage group	
I	Reference
II	0.1788
III	0.8649
IV	1.3601
Model 3	
Age	0.0185
ENSAT stage group	
I	Reference
II	0.1750
III	0.9343
IV	1.5587

*SEER* the Surveillance Epidemiology, and End Results database, *AJCC* the American Joint Committee on Cancer, *ENSAT* European Network for the Study of Adrenal Tumor

**Table 4:** Performance of models in the SEER training set

Models	C-index (95% CI)	P*
Model 1	0.715 (0.679–0.751)	–
Model 2	0.697 (0.660–0.734)	< 0.001
Model 3	0.698 (0.662–0.734)	< 0.001

*P values were obtained by comparing model 1 with model 2 and model 3, respectively

### Validation of the nomogram

The favorable discrimination ability of the nomogram was validated in the SEER internal validation dataset (C-index [95% CI], 0.672 [0.637–0.707]). In addition, the performance was also confirmed in the TCGA and Chinese multicenter external validation set, with C-indices of 0.810 (95% CI 0.732–0.888) and 0.726 (95% CI 0.633–0.819), respectively. Good consistency was also observed between actual survival data and the nomogram prediction in the three validation datasets (Fig. 2c–e). Therefore, the presented nomogram performed well in both the training and validation sets.

### Survival risk classification based on the nomogram

**Fig. 3:**
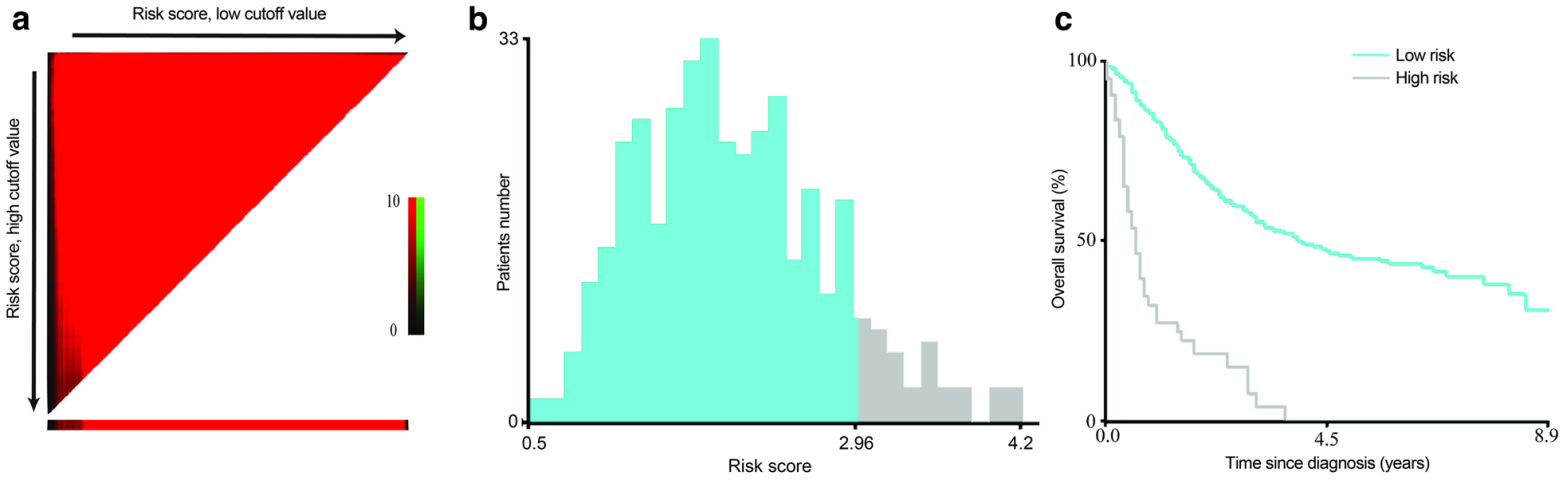
X-tile plots to identify the optimal risk score cutoff based on OS in the SEER training set. **a** X-tile plot for the training set. The X-tile plot was generated by dividing risk scores into three populations (low, middle and high) or two populations (low and high). Each pixel (point) of the X-tile plot represents the data from a given set of divisions. The X-axis represents all potential risk score cutoff from low to high (left to right) that define a low subset, whereas the Y-axis represents risk score cutoff value from high to low (top to bottom), that define a high subset. The arrows represent the direction in which the low subset (X-axis) and the high subset (Y-axis) increase in size. Data along the hypotenuse represent results from a single cutoff value that divides the data into high or low subsets. The coloration of the plot represents the strength of the association at each division, ranging from low (dark, black) to high (bright, red or green). Inverse associations between the risk score and survival are colored red, whereas direct associations are colored green. **b** The distributions of the number of patients by risk score. **c** Kaplan–Meier plots categorized by the low-risk and high-risk groups according to the optimal risk score cutoff. The optimal OS risk score cutoff was determined as 2.96 (χ^2^ = 97.7, *P* < 0.001)

**Fig. 4:**
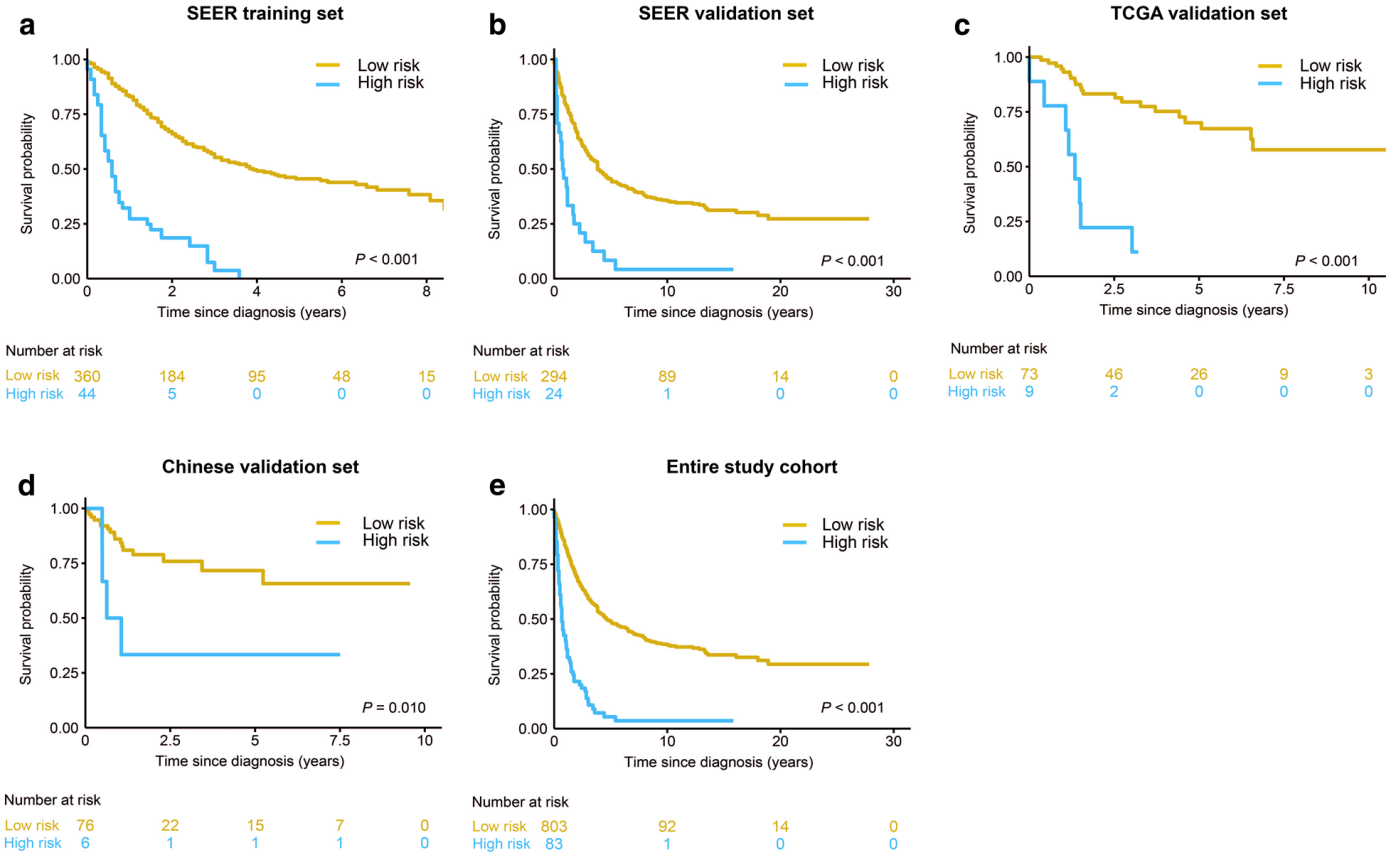
Kaplan–Meier survival curves categorized into low-risk and high-risk groups. Kaplan–Meier survival curves of OS in **a** training, **b** SEER internal validation; **c** TCGA validation; **d** Chinese multicenter validation set; **e** entire cohort of enrolled ACC patients. *OS* overall survival, *ACC* adrenocortical carcinoma, *SEER* the Surveillance Epidemiology, and End Results database, *TCGA* the Cancer Genome Atlas set

**Fig. 5:**
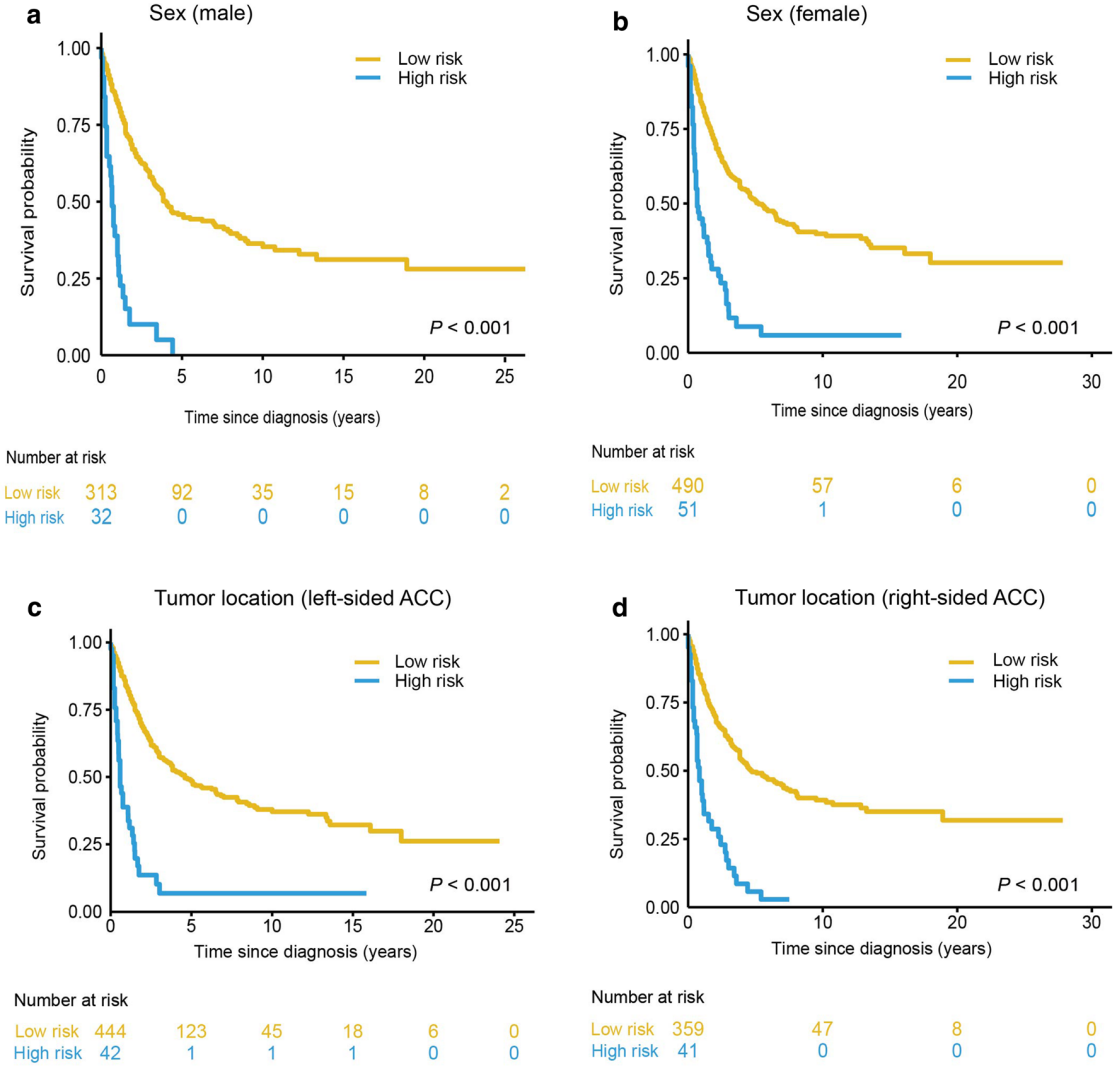
Kaplan–Meier survival curves categorized into low-risk and high-risk groups in stratified analyses for the entire study cohort. Significance between the OS of the high-risk and low-risk patients was observed in both sex **a** male and **b** female, and tumor location, **c** left-sided ACC, **d** right-sided ACC. *OS* overall survival, *ACC* adrenocortical carcinoma

The X-tile plots showed that the optimal mortality risk score cutoff value was 2.96 (Fig. 3), and was used to classify the patients into a high- (risk score ≥ 2.96) and low-risk group. Kaplan–Meier curves for survival outcomes of the different risk subgroups showed significant distinction in survival probability in the training set (Fig. 4a, *P* < 0.001); which was further confirmed in the three validation datasets (Fig. 4b–d, SEER internal validation set, *P* < 0.001; TCGA validation set, *P* < 0.001; Chinese multicenter validation set, *P* = 0.010). Further, the nomogram demonstrated great potential in distinguishing patients with high-risk of all-cause mortality in all the 886 investigated patients (Fig. 4e, *P* < 0.001) and the stratified analyses (Fig. 5). For the entire cohort, the median OS of patients in the low- and high- risk groups was 55.0 months (95% CI 43.1–67.1) and 8.0 months (95% CI 5.6–10.4), respectively.

### Clinical usefulness of the nomogram

**Fig. 6:**
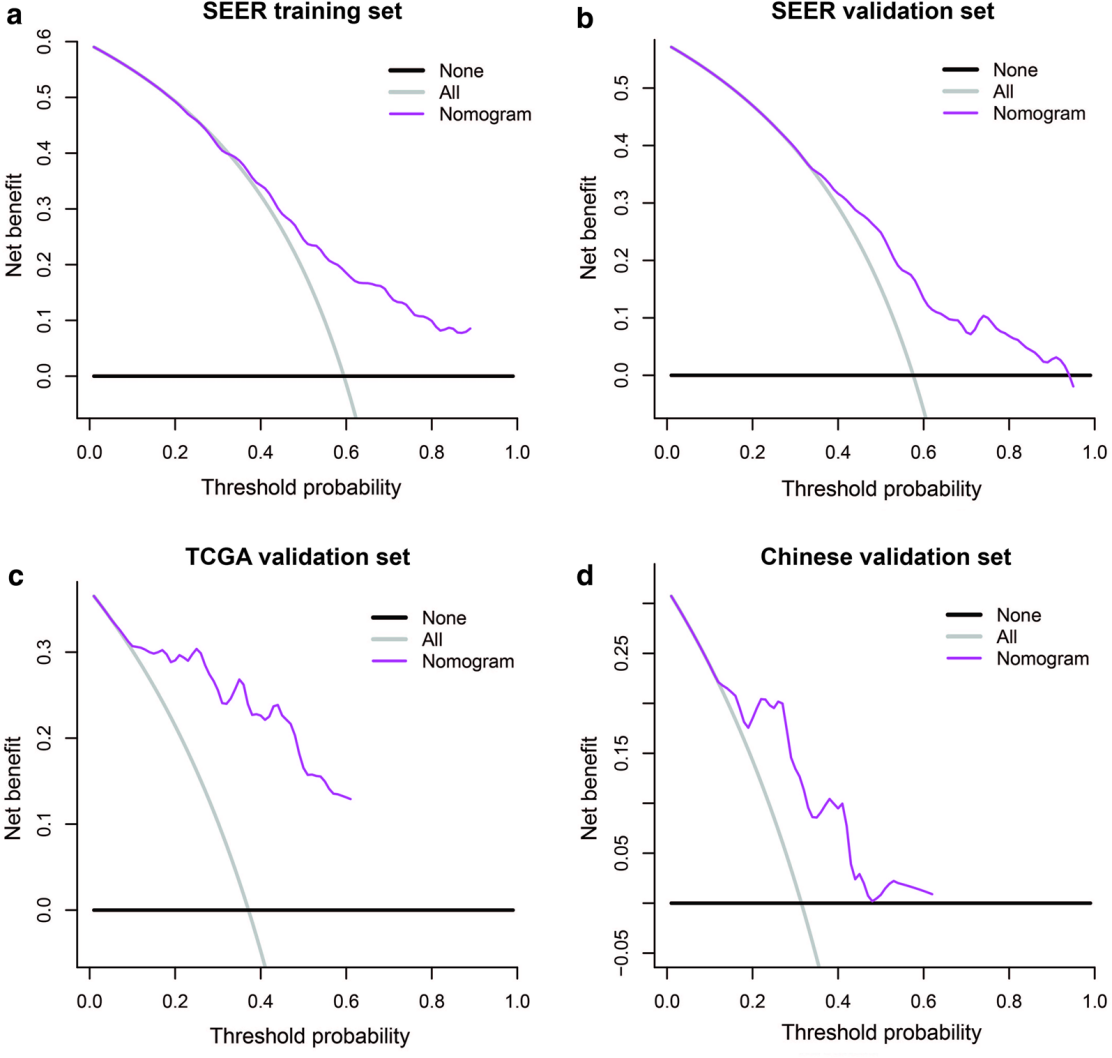
DCA for the nomogram. Decision curve analyses depicting the clinical net benefit in the different cohorts, namely the **a** SEER training; **b** SEER internal validation; **c** TCGA validation; **d** Chinese multicenter validation set. The horizontal solid black line represents the assumption that no patients will experience the event, and the solid gray line represents the assumption that all patients will relapse. On decision curve analyses, the nomogram showed superior net benefit in all different cohorts across a range of threshold probabilities. *DCA* decision curve analysis, *SEER* Surveillance Epidemiology, and End Results database, *TCGA* the Cancer Genome Atlas set

DCA analysis was performed to illustrate the net benefit at 5 years in each cohort. When the threshold probabilities exceeded 21% in the SEER training set, ranged between 34% and 95% in the SEER internal validation set, exceeded 6% in the TCGA validation set and 12% in the Chinese multicenter validation set, the use of the nomogram to predict the prognosis of adult ACC patients provided greater net benefit than the “treat all” or “treat none” strategies, indicating the favorable potential clinical applicability of the nomogram (Fig. 6).

## Discussion

In the present study, a nomogram incorporating age at diagnosis, T stage, N stage, and M stage was developed to predict the OS probability for adult ACC patients after surgery and was externally validated using multiethnicity and multicenter datasets. The nomogram showed good discrimination and calibration in both the training and validation datasets. Also, the DCA revealed it had promising clinical applicability. Thus, the constructed nomogram can provide an easy-to-use and individualized tool to help physicians to make more informed treatment decisions for treating adult ACC patients.

Concerning the development of ACC, the only curative approach to ACC is complete tumor resection. However, the 5-year survival after surgery for ACC range between 16 and 60% [22, 31, 32], showing the prognostic heterogeneity associated with this disease. Some studies reported that adjuvant therapy in localized disease may provide survival benefits [10, 33–35]. However, the necessity of adjuvant therapy remains elusive. Therefore, accurate prognostic predication after surgery for adult ACC patients is significant not only for the adjuvant treatment selection but also to inform patients about their long-term prognoses. However, there lacked a clear optimal method in present literature to predict the outcome of ACC patients and stratify them into different risk subgroups.

Several, but debatable, factors related to ACC prognosis were identified in previous studies. Some have reported that there were no correlations between age, sex, tumor size to the outcome of ACC [10, 36, 37], while others showed that age, sex, high tumor grade, and tumor size were significantly associated with prognosis [18, 31, 38]. Indeed, all of them focused on ACC patients of all ages (adults and pediatrics). The number of pediatric ACC patients was roughly about 12% to 20% of the total investigated cohort from these studies. Therefore, different biological behavior and clinical presentations between adult and pediatric ACC patients might have accounted for these inconsistent results. In contrast, in this present study, only adult patients were investigated and we found that age at diagnosis, T stage, N stage and M stage were independent predictors of OS after surgery. Similar to our findings, there have been other studies reporting that old age was a poorer prognostic factor for OS in adults as compared to the young patients [14, 36, 39]. OS might be affected by age not only related to the clinical course of the disease, but also for age-related complications [40]. Notably, a recent report has proposed a novel staging system incorporating patients’ age and was not based on the patient’s tumor size [14], because age at diagnosis may better inform clinicians about proper individualized treatment and prognostication. Also, in this present study, the TNM stage contributed as a main part of the final risk score and demonstrated better prognostic performance when combined age. Our nomogram had superior prognostic ability compared to the AJCC and ENSAT stage group models (Table 4). Also, its discrimination and calibration displayed good performance and was validated using internal and external validation datasets. Thus, it has the potential to be implemented in real-world clinical practice.

Further, the formulated nomogram can be comprehensively used for individualized treatment planification due to its potential to accurately stratify adult ACC patients based on their mortality risk [5, 8, 41] into two distinct prognostic groups, namely high- and low-risk groups. To the best of our knowledge, this is the first nomogram for predicting the OS of adult ACC patients after surgery. Compared with other prognostic models, our model was validated in three independent validation cohorts with promising results. The favorable discriminating ability of the nomogram in all validation sets supports its generalizability for routine clinical use.

Some limitations of the present study were as follows. First, this study may be potentially limited due to its retrospective nature and associated with inherent biases. We excluded patients with missing data during data collection as their inclusion would have simultaneously affected the credibility of the results. Second, the multivariable model did not include some potential prognostic predictors, such as the hormone status, Ki-67 index, Weiss score, SF-1, calretinin, and SRC1, because these informations were not uniformly available in the retrieved datasets. A more comprehensive model considering all potential risk factors might be expected to have better prognostic performance. Third, the follow-up time was shorter in the Chinese multicenter validation dataset, and close monitoring and five-year follow-up data are still required for these patients.

## Conclusions

In conclusion, we have developed a nomogram able to predict the postoperative OS tailored for adult ACC patients. The nomogram demonstrated favorable predictive accuracy and clinical usefulness after validation in datasets comprised of different populations and ethnicity. The proposed nomogram is an easy-to-use tool with promising clinical applicability to provide individualized patient counseling, timely surveillance, and clinical assessments.

## Data Availability

All data generated or analyzed during this study are included in this published article and its additional information files.

## Abbreviations

ACC: adrenocortical carcinoma
AIC: Akaike’s Information Criterion
AJCC: the American Joint Committee on Cancer
C-index: concordance index
DCA: decision curve analysis
ENSAT: the European Network for the Study of Adrenal Tumors
IQR: interquartile range
OS: overall survival
SEER: the Surveillance Epidemiology, and End Results
TCGA: the Cancer Genome Atlas
